# Integrating Protein Structure Prediction and Bayesian Optimization for Peptide Design

**DOI:** 10.21203/rs.3.rs-4045284/v1

**Published:** 2024-03-11

**Authors:** Negin Manshour, Fei He, Duolin Wang, Dong Xu

**Affiliations:** University of Missouri, Columbia, Columbia MO 65211, USA; University of Missouri, Columbia, Columbia MO 65211, USA; University of Missouri, Columbia, Columbia MO 65211, USA; University of Missouri, Columbia, Columbia MO 65211, USA

**Keywords:** Bayesian Optimization, Peptide Design, Deep Learning, Protein Structure Prediction

## Abstract

Peptide design, with the goal of identifying peptides possessing unique biological properties, stands as a crucial challenge in peptide-based drug discovery. While traditional and computational methods have made significant strides, they often encounter hurdles due to the complexities and costs of laboratory experiments. Recent advancements in deep learning and Bayesian Optimization have paved the way for innovative research in this domain. In this context, our study presents a novel approach that effectively combines protein structure prediction with Bayesian Optimization for peptide design. By applying carefully designed objective functions, we guide and enhance the optimization trajectory for new peptide sequences. Benchmarked against multiple native structures, our methodology is tailored to generate new peptides to their optimal potential biological properties.

## 1 Introduction

Modern pharmaceutical research finds drug discovery resource-intensive and time-consuming [1, 2]. Streamlining this process has profound economic and societal implications. A key phase involves identifying compounds that effectively bind to target proteins [3]. Recently, peptides, short chains of amino acids typically comprising fewer than 20 residues, have received more and more attention as potential binding candidates due to their biochemical traits and strong affinity [4]. Traditional experimental methods for peptide design are laborious [5, 6]. Computational techniques can often help experimental methods select target peptides more effectively and efficiently [7, 8]. In recent years, deep generative models have become pivotal in identifying peptide candidates, often bypassing traditional computational methods [9, 10, 11, 12, 13]. In particular, merging deep learning models with Bayesian optimization (BO) can further enhance sequence optimization [14, 15]. BO uses surrogate models like Gaussian Processes to approximate costly objective functions, balancing exploration and exploitation [16]. Integrated with deep learning, it enhances molecular discovery by prioritizing promising and uncertain candidates through model estimates and evaluations.

Since protein sequences are the primary input, we utilized deep learning models to translate these discrete sequences into continuous latent embedding needed for BO to refine the sequences. While most prior methods emphasized sequence-level insights, they often overlooked the importance of structure-level considerations. This oversight meant that the integration of protein-peptide structural nuances was often absent in their optimization strategies. In the wake of advancements by tools like AlphaFold2 [17], the prediction of protein-peptide complex structures has been significantly enhanced. Given that the 3D structures of protein-peptide complexes inherently carry vast amounts of information regarding their biochemical functionality, there is an opportunity to refine objective functions grounded in 3D complex structures.

In this study, we started from Lambo [14], one of the sequence-based methods, which melds BO with denoising autoencoders for advanced protein sequence design using a multi-objective Gaussian surrogate process. We complemented Lambo with the protein modeling capabilities of ColabFold [18] due to its speed in comparison with AlphaFold2, enabling quicker iterations to predict the complex structures of generated peptides and target proteins. Additionally, we incorporated specific scoring functions that scrutinize these complex structures, further enhancing the optimization of peptide sequences. By doing so, we can ensure the selection and optimization of peptides are not only high-quality but also exhibit desired interactions when complexed with target proteins.

## 2 Methodology

This study introduces a model that synergizes ColabFold and the BO process to craft peptides with specific properties. During each iteration, new peptide sequences were assessed by establishing objective functions for their predicted complex structures in conjunction with the target protein. All generated optimized peptide sequences in each iteration are subsequently reintroduced to the candidate pool for passing the optimization process. To validate our method, we selected native protein-peptide complexes in PDB and artificially mutated the peptides. A protein sequence and the corresponding mutated peptide sequence were fed into our model, and we evaluated to what extent our model could recover the native protein-peptide complex.

### 2.1 Dataset and Data Preprocessing

During our research’s outset, we prioritized data preprocessing for subsequent stages. We obtained protein-peptide structures from the PDB website to form our benchmark dataset. Native peptide sequences were artificially mutated for optimization seeds. Executing a BLAST on these sequences yielded mutated variants in natural selection, broadening our analysis pool. This pool, comprised of base seeds and optimized sequences from each round, is combined with previous seeds to furnish data for the next round, ensuring a comprehensive and evolving dataset for analysis. This thorough preparation readied sequences for the optimization process.

### 2.2 Algorithm

**Figure 1:**
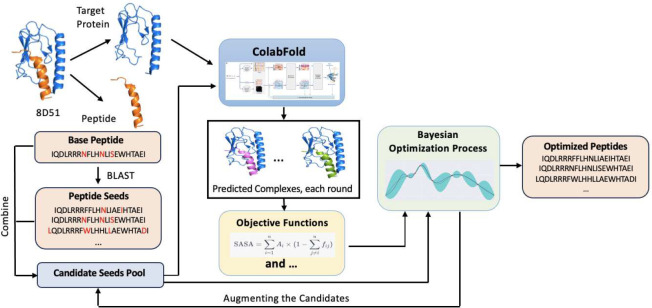
Architecture illustrating the integration of ColabFold and BO [21] framework. In the data preprocessing phase, seeds are amalgamated to form a comprehensive candidate pool. Protein-peptide complex structures are then predicted for all peptides with a consistent target protein. By obtaining objective scores for these structures, the necessary input data is readied for the BO section, facilitating peptide sequence optimization.

Our algorithm for peptide design is illustrated in Figure 1. We harnessed the power of BO, capitalizing on its ability to operate on latent embeddings of peptides. First, a non-autoregressive denoising autoencoder converts discrete sequences into continuous token-level embeddings using Lambo, creating a representation that captures the inherent characteristics and patterns of the peptide sequences. By operating on latent embeddings, and continuous representations of discrete sequences, BO efficiently traverses the vast sequence landscape. Central to BO is the Noisy Hypervolume Improvement (NEHVI) acquisition function [19], an extension of the Noisy Expected Improvement [20] tailored to multiple objectives that balance exploration and exploitation. The acquisition function guides the search to promising regions in the latent space. This iterative process, underpinned by latent embeddings, ensures convergence towards sequences with desired properties. Furthermore, the probabilistic nature of BO provides a measure of uncertainty, aiding in decision-making. By leveraging these embeddings, BO offers a nuanced, data-efficient approach to complex sequence optimization tasks.

In our current implementation, we have structured the optimization process to encompass 64 rounds, with each round consisting of a batch size of 16 peptide sequences. This systematic approach allows us to evaluate and mutate these sequences iteratively. As a result, in every round, we are able to extract 16 distinct mutated peptide sequences. This methodological design ensures a comprehensive and thorough exploration of the sequence space, facilitating the identification of optimized peptide variants with desired properties.

### 2.3 Objective Functions

Objective functions are key for evaluating our peptide sequences. For every sequence, we predicted complex structures with the target protein for further analysis. The first function uses the “Solvent Accessible Surface Area” (SASA) [22] algorithm to assess the interaction strength between the peptide and protein. The second evaluates the stability of the complex structure. The third examines the binding sites between the peptide and the protein by measuring the distance between residues; residues closer than 5Å are considered binding sites. The ratio of these sites gives insight into the interaction’s strength. Together, these functions create a solid framework in ColabFold for peptide sequence optimization.

#### 2.3.1 Solvent Accessible Surface Area (SASA)

We calculate the Solvent Accessible Surface Area (SASA) of the protein-peptide complex using the Shrake and Rupley algorithm [23, 24]. The calculation is performed at the atomic level, using a probe radius of 1.4 Å to approximate the solvent molecule. We employed a discrete number of points to represent the surface area of each atom and then estimated the SASA by subtracting the SASA of the complex from the combined SASAs of the individual components, protein, and peptide. SASA value indicated the interaction between protein and peptide based on the mutations in the peptide sequence. Since mutated peptides have different accessible surfaces. Our modified approach for computing the Solvent-Accessible Surface Area (SASA) diverges from the original LaMBO version by focusing on the binding region between the protein and peptide. Unlike the traditional method, which quantifies the solvent-accessible surface of an isolated protein, our technique evaluates the interface where the protein and peptide interact. This tailored assessment provides specific insights into the binding interactions, rather than the general solvent exposure of the protein alone.

#### 2.3.2 Energy

Our methodology involves optimizing peptide sequences, guided by their predicted structures, to enhance stability, as indicated by negative changes in Gibbs free energy (-dG). The evaluation of energy using FoldX [25] has demonstrated a significant correlation with peptide functionality and stability. Consequently, this parameter was adopted as a critical objective function in our optimization process. Lower Gibbs free energy signifies greater stability, making it a key indicator of improved protein-peptide interaction in our methodology.

#### 2.3.3 Binding Sites Ratio

Our method quantifies the interactions between a peptide and a protein using calculated binding ratios. This assessment hinges on the spatial proximity of residues within a specified cutoff distance, set at 5Å [26] for our current tests. The binding ratio within the protein is ascertained by the proportion of unique protein residues that are in proximity to any peptide residue, compared to the total number of residues in the protein chain. In a similar vein, the binding ratio for the peptide is calculated.

This methodology provides a detailed perspective on molecular interactions, which is important for elucidating the binding dynamics and stability in protein-peptide complexes. In our optimization process, we can select any two of these parameters as objective functions in the code settings. This enables us to rigorously evaluate each newly generated peptide, ensuring a continuous improvement in our understanding and optimization of peptide-protein interactions. Additionally, once all iterations of the optimization process are completed, we can extract all desired optimized peptides based on their objective function values. This extraction at the end of the process allows for a comprehensive evaluation and selection of the most promising peptides.

## 3 Evaluating optimized peptides

**Figure 2:**
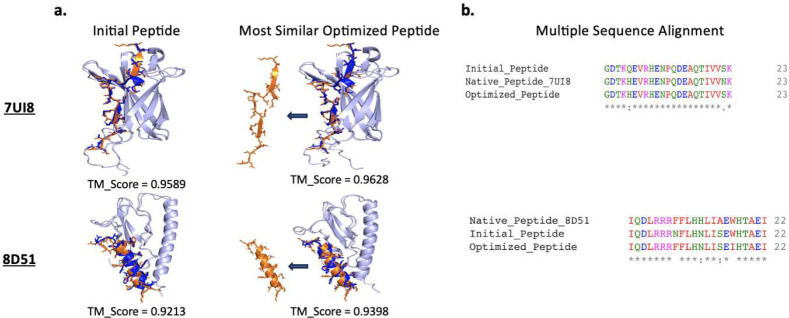
The qualities of optimized peptides. a. Calculation of the TM-score for the initial peptide sequence and the sequence optimized to be most similar to the native one. In this figure, the native and predicted protein structures are represented in light blue, the native peptide in dark blue, and the most similar optimized peptides are in orange. b. The corresponding Multiple Sequence Alignment is shown for two (initial and most similar) peptide sequences and the native sequences.

After progressing through specific iterations, notably round=53 for 7UI8 and round=15 for 8D51, we have successfully identified sequences strikingly close to their native counterparts. Based on the structural predictions, shown in Figure 2a,2b, the TM scores for both samples are impressive. This underscores our method’s ability to maintain the essential structure of the complex while the peptide sequences undergo modifications and optimizations throughout the process.

**Figure 3:**
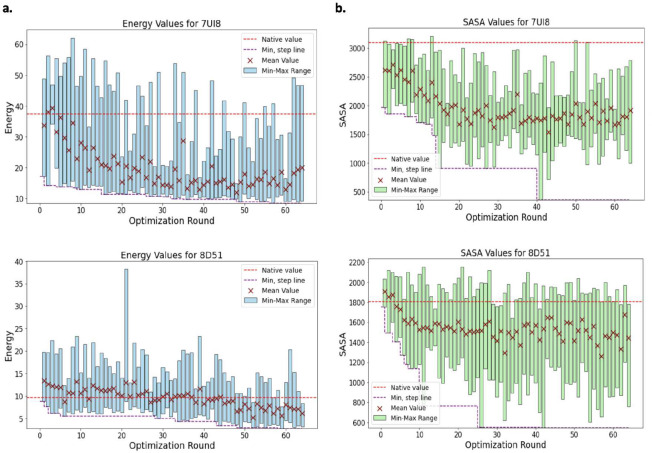
Optimization results of energy and SASA. a and b. Bar charts show the trends of the minimum-maximum and the average value of energy and SASA over the optimization rounds for both samples. In each plot, the least values of energy and SASA over all rounds are also indicated. The values are compared to the corresponding energy and SASA native values.

As depicted in Figure 3a,3b, a consistent trend is observed across the optimization rounds for both samples. With the progression of rounds, there is a noticeable decline in both SASA and energy values. This trend underscores the efficiency of the optimization process; as the rounds increase, the sequences are directed toward enhanced stability and improved binding affinity. Specifically, 41.86% of sequences from 7UI8 and 12.01% from 8D51, across all optimization rounds, match the number of binding sites found on the native peptide in the complex, indicating values consistent with their native counterparts.

## 4 Conclusion

In conclusion, our innovative approach, combining BO with protein modeling, shows great promise in peptide design. The results highlight its success in generating new peptides that bind well with target proteins and have useful biological functions. Traditional methods, mainly focusing on sequence properties, often miss the complexities of peptide design. Our approach fills this gap by incorporating predicted complex structures into the evaluation process, supported by carefully designed objective functions. This ensures the structural features of peptide sequences are maintained and optimized when combined with target proteins. This method has some limitations, such as the extensive computation time required. Additionally, the current embedding method for protein sequence may not be ideal, suggesting a potential area for future exploration and improvement in embedding techniques. We will investigate better algorithms and embedding methods to improve the method’s efficacy.
